# A26 BMP-SIGNALING IMPAIRED TELOCYTES CREATE A DISRUPTED NICHE GRADIENT FOSTERING COLITIS-ASSOCIATED CANCER

**DOI:** 10.1093/jcag/gwac036.026

**Published:** 2023-03-07

**Authors:** V Reyes, V Pomerleau, P Granger, N Perreault

**Affiliations:** 1 Département d'Immunologie et de Biologie Cellulaire; 2 Département de pathologie, Universite de Sherbrooke, Sherbrooke, Canada

## Abstract

**Background:**

The colonic stem cell niche is established by a gradient of WNT, R-spondin, BMP factors and their antagonists along the colonic epithelial vertical axis. Telocytes (TC^FoxL1+^) are mesenchymal cell forming a 3D hub underneath the epithelium, identified as an important source of niche factors. Specifically, they express non-canonical (nc) WNT factors and are the richest source of BMPs. Disruption of the BMPs gradient has been shown to be related to the development of several gastrointestinal diseases like Inflammatory Bowel Diseases (IBD). Such chronic inflammation drives the onset of Colitis-Associated Cancer (CAC) in about 60% of IBD patients. We previously showed that following a chronic inflammatory stress, 50% of the KO mouse for the BMP receptor 1a in colon telocytes (*Bmpr1a*^△FoxL1+^) presented malignant epithelial transformations. These cancer-like regions showed an aberrant epithelial b-catenin localization and an enlargement of the double positive α-SMA^+^/Vimentin^+^ mesenchymal population.

**Purpose:**

Loss of BMP signaling in TCFoxL1+ affects the mesenchymal-epithelial crosstalk and makes the colonic epithelium vulnerable to injuries, promoting/perpetuating inflammation fostering CAC onset.

**Method:**

Following a DSS-based chronic inflammatory challenge in mutant and control mice, TCFoxL1+ ultrastructure was analyzed using transmission electron microscopy. Expression levels of members of the WNT-BMP axis (BMPs, WNTs and associated antagonists) were evaluated by qPCR in tumor-like areas and adjacent tissue. YAP cellular localization was evaluated by immunofluorescence in colon after chronic DSS challenge in *Bmpr1a*△FoxL1+ mice and controls. To differentiate cancer-associated fibroblasts (CAFs) subtypes, myCAF (myofibroblastic) and iCAF (inflammatory), in tumor-like region and adjacent tissue, we used co-staining against gp38, ICAM, Tagln and αSMA.

**Result(s):**

Following a chronic DSS-challenge, electron microscopy analysis demonstrated that TC^FoxL1+^ in the control mice exhibited a shortening and erosion in their telopodes (Tp). TC^FoxL1+^ in *Bmpr1a*^△FoxL1+^ mice tumor-like regions presented an expanded endoplasmic reticulum with fragmented and dilated Tp. A significant increase in BMP 4, 5 and 7 and in Wnt5 (nc) was detected in *Bmpr1a*^△FoxL1+^ mice compared to controls. Confocal analysis revealed a strong nuclear accumulation of YAP in cancer-like regions in mutant mice compared to controls. Finally, tumour-like regions presented an heterogeneous distribution of iCAF and myCAF compared to controls.

**Conclusion(s):**

These results exposed that the disruption of TCFoxL1+ associated BMP signaling disturbs the WNT-BMP gradient essential for the optimal maintenance of the SC niche and thus impacting epithelial regeneration when under stress. Thus, defective TCFoxL1+ assume a key role in the poor regeneration process of the epithelium which in the end promotes the development and progression of CAC.

**Please acknowledge all funding agencies by checking the applicable boxes below:**

CIHR

**Disclosure of Interest:**

None Declared

